# CETD, a global compound events detection and visualisation toolbox and dataset

**DOI:** 10.1038/s41597-025-04530-x

**Published:** 2025-02-28

**Authors:** Cong Yin, Mingfang Ting, Kai Kornhuber, Radley M. Horton, Yaping Yang, Yelin Jiang

**Affiliations:** 1 Institute of Geographic Sciences and Natural Resources Research, Chinese Academy of Sciences, Beijing, China; 2 Lamont-Doherty Earth Observatory, Columbia University, Palisades, NY USA; 3 Sierra Nevada Research Institute, University of California, Merced, CA USA; 4 Columbia Climate School, Columbia University, New York, NY USA; 5 International Institute for Applied Systems Analysis, IIASA, Laxenburg, Austria; 6 Jiangsu Center for Collaborative Innovation in Geographical Information Resource Development and Application, Nanjing, China

**Keywords:** Climate-change mitigation, Climate-change impacts

## Abstract

Compound events (CEs) are attracting increased attention due to their significant societal and ecological impacts. However, their inherent complexity can pose challenges for climate scientists and practitioners, highlighting the need for a more approachable and intuitive framework for detecting and visualising CEs. Here, we introduce the Compound Events Toolbox and Dataset (CETD), which provides the first integrated, interactive, and extensible platform for CE detection and visualisation. Employing observations, reanalysis, and model simulations, CETD can quantify the frequency, duration, and severity of multiple CE types: multivariate, sequential, and concurrent events. It can analyse CEs often linked to severe impacts on human health, wildfires, and air pollution, such as hot-dry, wet-windy, and hot-dry-stagnation events. To validate the performance of CETD, we conduct statistical analyses for several high-impact events, such as the 2019 Australian wildfires and the 2022 European heatwaves. The accessibility and extensibility of CETD will benefit the broader community by enabling them to better understand and prepare for the risks and challenges posed by CEs in a warming world.

## Background & Summary

Communities worldwide are witnessing an increasing number of extreme weather and climate events due to enhanced greenhouse gas concentration in the atmosphere^1^. As an emerging climate hazard, compound events (CEs) can have significant impacts on multiple societal sectors, including human health, energy, agriculture, and the environment^2–4^. CEs refer to the combination of multiple climate drivers and/or hazards that contribute to social or environmental risks^5^. These drivers and hazards do not need to reach extreme levels to cause significant impacts^6,7^. Compared to individual extreme events, CEs involve complex spatial and temporal interactions of multiple climate hazards, often with impacts that exceed the sum of the individual occurrences of each hazard. The simultaneous or consecutive occurrence of extremes in temperature, precipitation, or wind can quickly overwhelm the resilience of human and natural systems^6^, resulting in significant societal and ecological impacts^8^. CEs may provide preconditions that lead to severe or catastrophic impacts, such as wildfires and coastal floods^9,10^. In June 2023, hot-dry events caused extensive wildfires in Canada, and the dense smoke made the northeastern United States one of the regions with the poorest air quality globally^11^. Another example is Typhoon Haiyan in November 2013 with over 6,000 lives lost in the Philippines, due to the close succession of heavy precipitation and storm surge that led to catastrophic flooding^12^. There is an increasing trend in multiple types of CEs, such as extreme winds and precipitation, heatwaves and droughts, and droughts and aridity^13,14^. This trend underscores the need for widely accessible tools to track newly occurring high-impact events. However, comprehensive tools for identifying CEs are rather limited, hindering efforts to facilitate risk assessment and adaptation planning by climate scientists and practitioners.

Compared to traditional multi-hazard frameworks, CE primarily considers drivers and hazards related to the climate system^15^. A typology proposed by Zscheischler *et al*. differentiates CEs into four types: preconditioned, multivariate, temporally compounding, and spatially compounding^6^. Multiple types of CEs within this framework have been investigated, often motivated by the societal and ecological impacts they entail. Ridder *et al*. conducted a comprehensive global analysis of CEs associated with floods, droughts, and wildfires, covering a total of 27 combinations of variables like temperature, precipitation, wind speed, hail, river flow, storm surge, and forest fires^8^. Li *et al*. showed that heatwaves are more likely to occur after snow droughts, which may be due to increased soil aridity and atmospheric drying following snow droughts^16^. Leeding *et al*. explored the dynamical mechanism between winter cold spells over North America and European wind extremes, as an example of spatially and temporally compound cold and windy events^17^. Several attempts have been made to expand CEs research into multi-hazard spheres by including variables beyond the climate system, such as floods, crop failures, and landslides^18^.

Previous studies have suggested several methods to identify CEs. (i) Simple superposition is a binary classification method that identifies CEs when multiple individual extreme events occur simultaneously^19^. However, this method does not quantify the severity of CEs. (ii) Joint probability treats multiple environmental factors as different random variables and detects CEs using the combined density distribution and a set of respective thresholds^20^. This method allows for the quantification of CEs’ occurrences conditioned upon a given severity measure. (iii) Spatial clustering detects CEs by grouping multiple individual extreme events within a certain spatiotemporal distance. This method can cluster spatiotemporally disjointed events, avoiding artificial fragmentation and enhancing extensibility in CEs’ detection. A representative example has been employed to identify compound wind and precipitation extremes in Great Britain, with potential for extension to other events^21^. Finally, (iv) Event encoding establishes a coding system for CEs’ detection, improving the efficiency of detecting CEs and enabling the determination of different types of CEs based on the coding system^22^. While existing studies have provided climate scientists and practitioners with a variety of analytical results, the inherent complexity of CEs still poses challenges for their detection, analysis, and intercomparison, highlighting the need for a more comprehensive effort, based on an approachable and intuitive framework supported by a credible set of datasets. Furthermore, existing analytical tools and datasets associated with CE characteristics are very limited, and the substantial computational effort required for data collection and preprocessing tasks has hindered researchers from advancing their studies on CE.

In this study, we propose a CE detection method, along with the first integrated and interactive toolbox for tracking CEs. The method follows the methodology of simple superposition, which detects CEs through spatiotemporal connectivity and identifies the types of CEs based on an event coding system. We developed the toolbox with a focus on accessibility and extensibility, capable of detecting and visualising the frequency, duration, and severity of CEs. A series of case studies based on the outputs of the toolbox suggests that recent high-impact events are strongly associated with CEs. We believe that CETD will not only provide tools and data to support new scientific discoveries but also offer technical support to the public, community, and private sector, among others. In the following sections, we present the three-step data production methodology (Methods) and analyse some high-impact events to demonstrate the performance of CETD (Technical Validation).

## Methods

**Fig. 1:**
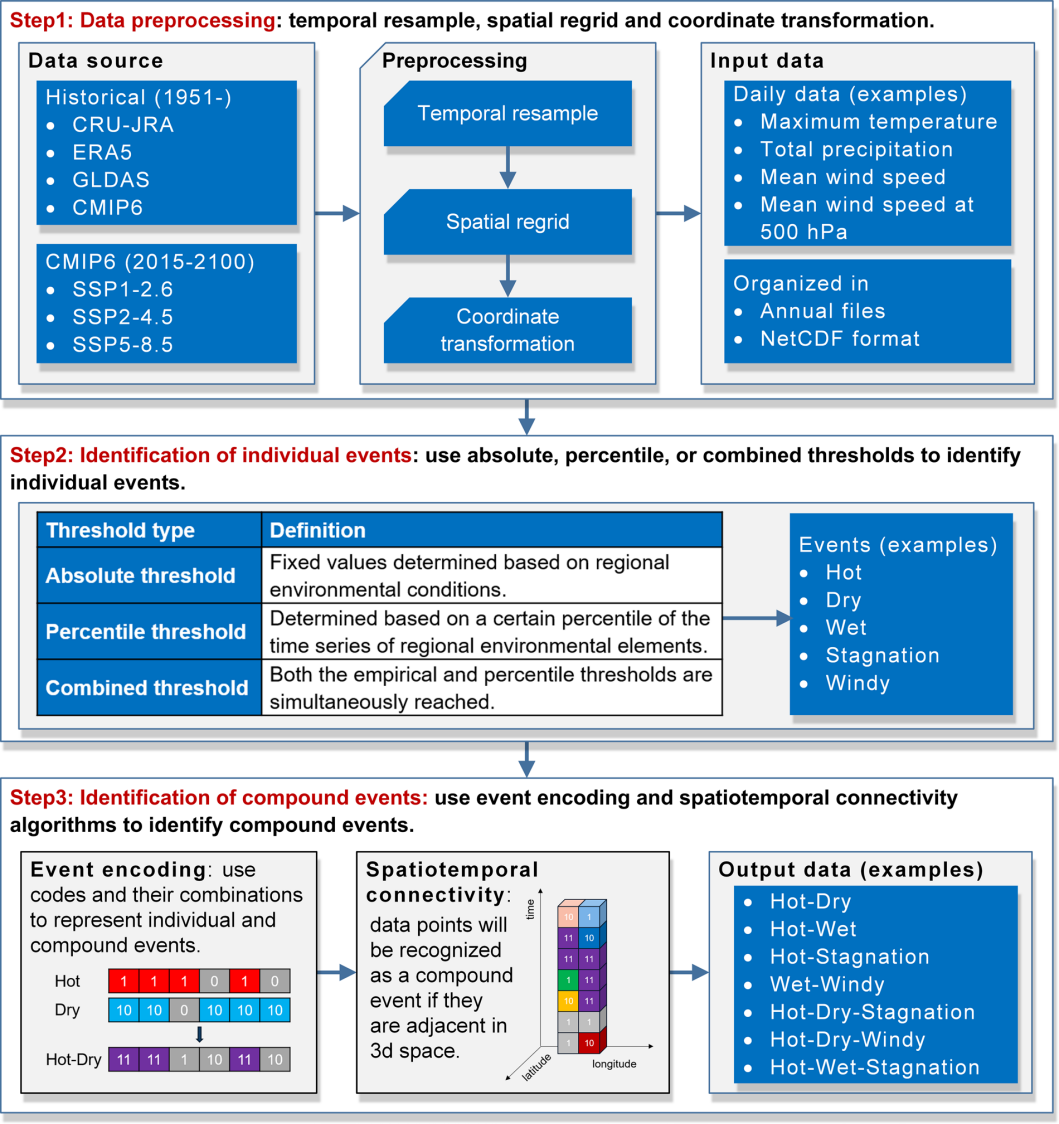
Technical flowchart of the data production consists of three steps: (i) data preprocessing, (ii) identification of individual events, and (iii) identification of compound events.

CETD uses event encoding and spatiotemporal connectivity algorithms to identify CEs. Event encoding involves the use of numbers to represent different CEs, and spatiotemporal connectivity allows the identification of multiple spatially and temporally connected grid points that meet the CE threshold as an individual CE, enabling the identification of CE type and extent, respectively. Figure 1 outlines the three-step procedure used to identify CEs, followed by detailed descriptions of data preprocessing, and individual and compound events identification.

### Data preprocessing

CETD supports multiple sources of input data that have been appropriately pre-processed (Fig. 1, step 1). First, the required input data are determined by the time, region, and CE types of interest. For example, to generate global hot-dry event records for 2022, global daily temperature and precipitation data for 1991–2020 (the reference period) and 2022 are needed. Second, the input data is processed to have consistent spatial resolution to perform calculations. For example, as input data, daily temperature and precipitation data are regridded to the same resolution, e.g., 0.5°. Finally, the input data is in NetCDF format containing daily climate variables for each year. For example, “tasmax_2022.nc” represents the daily maximum temperature (tasmax) for the year 2022.

In this study, we primarily focus our results based on the European Centre for Medium-Range Weather Forecasts (ECMWF) reanalysis v5 (ERA5) (Technical Validation section)^23,24^. For broader applications, we provide pre-processed daily climate data derived from ERA5, the Climate Research Unit Japan Reanalysis (CRU-JRA)^25^, and the Global Land Data Assimilation System (GLDAS)^26^. Additionally, we offer annual CE characterization data generated by CETD based on ERA5 and the Coupled Model Intercomparison Project Phase 6 Global Climate Models (CMIP6 GCMs)^27^ (Data Records section). The selection criteria for CMIP6 GCMs were based on the availability of daily maximum temperature, total precipitation, and mean wind speed during 1951–2014 (historical simulations) and 2015–2100 (SSP1 2.6, SSP2 4.5, and SSP5 8.5). CRU-JRA, ERA5, and GLDAS data were regridded to 0.5° using linear interpolation, while CMIP6 GCM data were regridded to 1° due to lower initial resolution.

### Identification of individual events

After data preprocessing, CETD provides absolute, percentile, and combined thresholds to identify individual events (Fig. 1, step 2). Absolute thresholds are fixed values based on regional environmental conditions. For example, one may use daily maximum temperature exceeding 35 °C to define extreme hot days. One drawback is that it is not possible to use the same threshold across the whole region due to differences in climatology across regions^22^. Local percentile thresholds are determined based on a certain percentile of a time series of regional environmental factors and have been widely used in large-scale studies^13,21^. Combined thresholds stipulate that extreme events can only be identified if both absolute and percentile thresholds are met^22^. The selection of the threshold methodology should consider the variable (e.g., temperature, precipitation, wind speed), the spatial scale (e.g., global, continental, national), and the affected sectors (e.g., health, agriculture, ecosystems).

This study focuses on five individual events that may lead to societal and ecological impacts. Among them, “stagnation” is defined by absolute thresholds following Horton *et al*.^28^, while “hot,” “dry,” “wet,” and “windy” events are defined by percentile thresholds. While the specific percentile thresholds inevitably vary among existing studies, they often exhibit consistency or complementarity across different components of CEs. For instance, Bevacqua *et al*. defined hot and dry events as temperatures above the 90th percentile and precipitation below the 10th percentile^29^. Beniston employed a similar approach but used the 75th and 25th percentiles^30^. For wet and windy events, Waliser *et al*. and Ridder *et al*. utilised precipitation and wind speed above the 98th and 99th percentiles, respectively^8,31,32^. Here, we use 1991–2020 as the reference period and utilise the 95th and 5th percentiles as the thresholds for defining high percentile (for hot, wet, and windy events) and low percentile (for dry events) respectively, hereafter 95th/5th. Leveraging the flexibility of CETD, we also present results based on the 90th/10th and 98th/2nd percentiles to demonstrate the sensitivity of the results to the selected thresholds (Technical Validation section and Supplementary Information). CETD calculates thresholds at the grid point level and provides a “sample_size” variable in its output file, which indicates the number of data points used for threshold calculation. The definitions of the individual events are detailed in Table 1.

**Table 1:** Definition of individual extreme events.

Extreme event	Definition
Hot	tasmax is above the 95th percentile of daily values for the season during the reference period.
Dry	pr is below the 5th percentile of daily values for the season during the reference period. Only days with pr > 0 mm are considered for calculating the threshold.
Wet	pr is above the 95th percentile of daily values for the season during the reference period. Only days with pr > 1 mm are considered for calculating the threshold.
Stagnation	sfcWind < 3.2 m/s and preWind500 < 13 m/s.
Windy	sfcWind is above the 95th percentile of daily values for the season during the reference period. Only days with sfcWind > 0.5 m/s are considered for calculating the threshold.

* tasmax: daily maximum temperature.

pr: daily total precipitation.

sfcWind: daily mean wind speed at near-surface level.

preWind500: daily mean wind speed at 500 hPa pressure level.

The reference period is 1991–2020.

### Identification of compound events

**Fig. 2:**
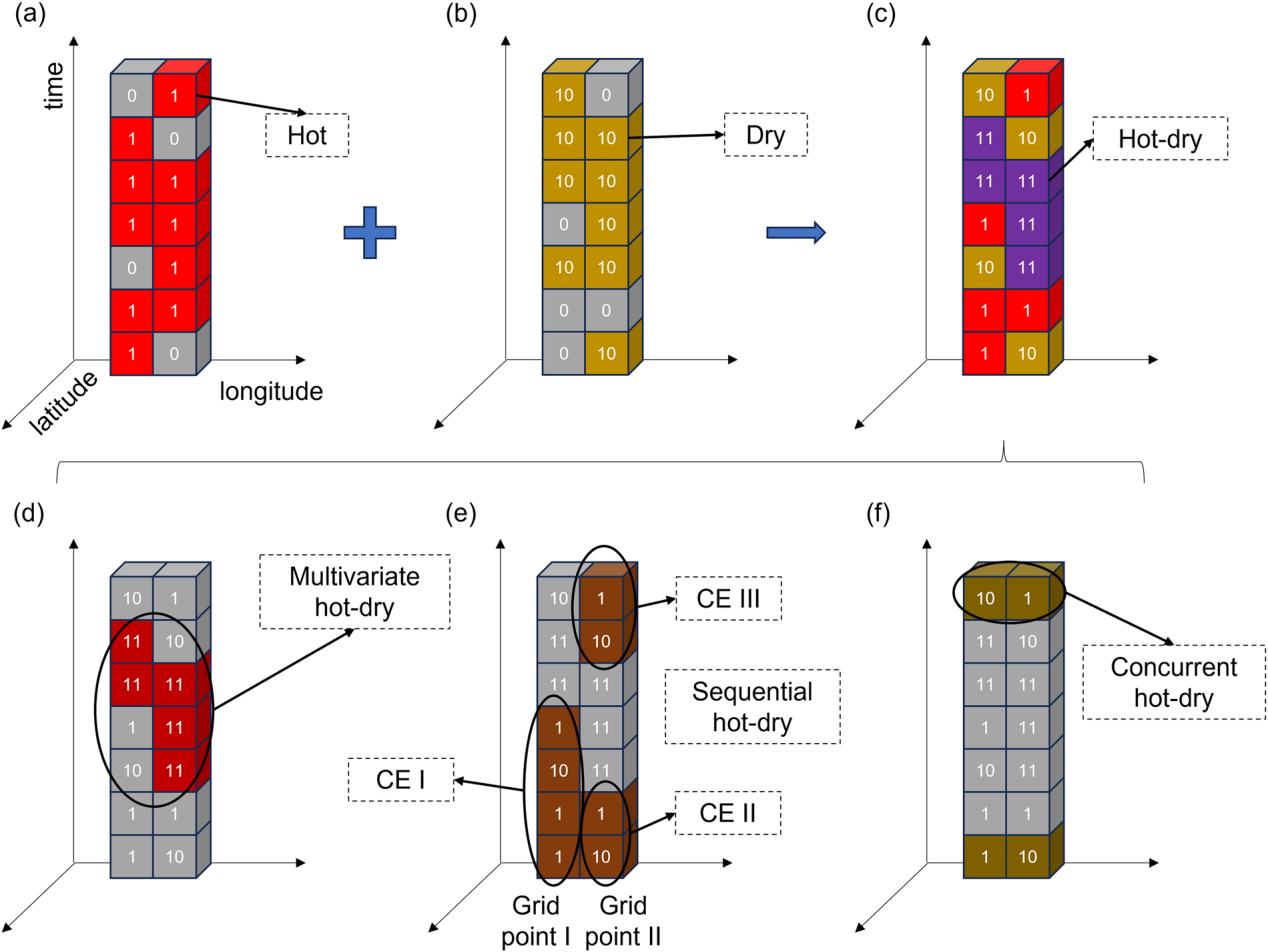
Schematic diagram of the event encoding and spatiotemporal connectivity algorithms for detecting individual events (**a,b**) and their combination (**c**), as well as multivariate events (**d**), sequential events (**e**), and concurrent events (**f**).

As stated earlier, CETD employs event encoding and spatiotemporal connectivity algorithms to identify CEs (Fig. 1, step 3). For instance, in Fig. 2, we use “1” to represent a hot event, “10” to represent a dry event, and their combination, “11,” to represent a hot-dry event. Furthermore, the spatiotemporal connectivity algorithm identifies adjacent grid points with the same numerical value as CEs. As depicted in Fig. 2c, the purple grid points labelled as “11” will be recognized as a hot-dry event.

CETD offers the capability to recognize three types of CEs: multivariate, sequential, and concurrent events. Multivariate events (same location and same time) refer to the simultaneous occurrence of two or more individual events of different variables at the same location (Fig. 2d). Sequential or temporally compounding events (same location over a period) involve multiple individual events of the same or different variables occurring successively at a location (Fig. 2e). Concurrent or spatially compounding events (different locations within the same time range) indicate the simultaneous occurrence of multiple individual events in different regions (Fig. 2f). CETD also provides parameters for investigating refined CEs such as persistent hot-dry events and rapid transition hot-dry events^33,34^. This is achieved by excluding multivariate events with too-short durations and sequential events with too-long intervals. Compared to the commonly used grid point-based methods in existing studies, CETD significantly improves the flexibility and efficiency in detecting CEs.

The output of CETD includes multiple characteristics of CEs, as shown in Table 2. Among them, the definition of the number of events, number of days, duration, and start/end dates of CEs are grounded in previous research^35,36^. For the severity of events, we use aggregated anomalies of climate variables involved in CEs relative to the reference period (1991–2020). For example, the average/peak severity of boreal summer hot-dry events (June, July, and August) is determined by identifying the average/maximum severity among all days that meet the hot-dry thresholds during the summer season. The severity of each day is calculated as the average standardized anomaly of temperature and precipitation relative to the daily values during the summers from 1991 to 2020. In Fig. 2e, for example, Grid point I has one CE (CE I), and Grid point II has two CEs (CE II and CE III), with the number of events being 1 and 2, respectively. The durations of CE I, CE II, and CE III are 4 days, 2 days, and 2 days, so the total number of days exceeding the threshold for both Grid point I and Grid point II is 4 days. The start date for Grid point I is the first day, and the end date is the fourth day. For Grid point II, the start date is also the first day (start of the first CE), and the end date is the seventh day (end of the last CE). The average and peak severity of Grid point I and Grid point II are determined by the average and maximum anomaly, respectively, across the four days that exceed the CE threshold.

**Table 2:** Definition of CEs’ characteristics.

Characteristic	Definition
Number of events	The number of CEs on each grid point. Reaching the threshold on consecutive days will be counted as one CE.
Number of days	The total number of days the threshold was reached on each grid point.
Maximum (Minimum) duration	The longest (shortest) number of consecutive days the threshold was reached for a CE on each grid point.
Average duration	The average of the durations of all CEs at each grid point, calculated as the number of days divided by the number of events.
Average (Peak) severity	Average (maximum) aggregated anomalies of climate variables involved in CEs relative to the reference period on each grid point.
Start (End) date	The start (end) date of the first (last) CE on each grid point.
Extent	The proportion of land area within a region that has experienced at least one CE.

We identified seven combinations of multivariate events that have the potential to cause significant societal and ecological impacts. Among these combinations, the adverse effects of hot-dry events on human health, food security, ecosystem productivity, and supply chains have been extensively studied^37–40^. Additionally, hot-dry-stagnation and hot-dry-windy events are also considered due to the exacerbating effects of stagnation and high wind speeds on air pollutant concentrations and the spread of wildfires, respectively^41^. Hot-wet, hot-stagnation, and hot-wet-stagnation events are associated with human perception of ambient temperature, as high humidity and low wind speeds impede sweat evaporation and heat dissipation from the skin, thereby increasing the risk of heat-related illnesses such as heat cramps, heat exhaustion, and heatstroke^42^. Wet-windy events, characterized by heavy rainfall and strong winds, can lead to overland flooding and storm surges, resulting in compound flooding with severe impacts on coastal livelihoods^43^.

### Compound Events Toolbox and Dataset, CETD

**Fig. 3:**
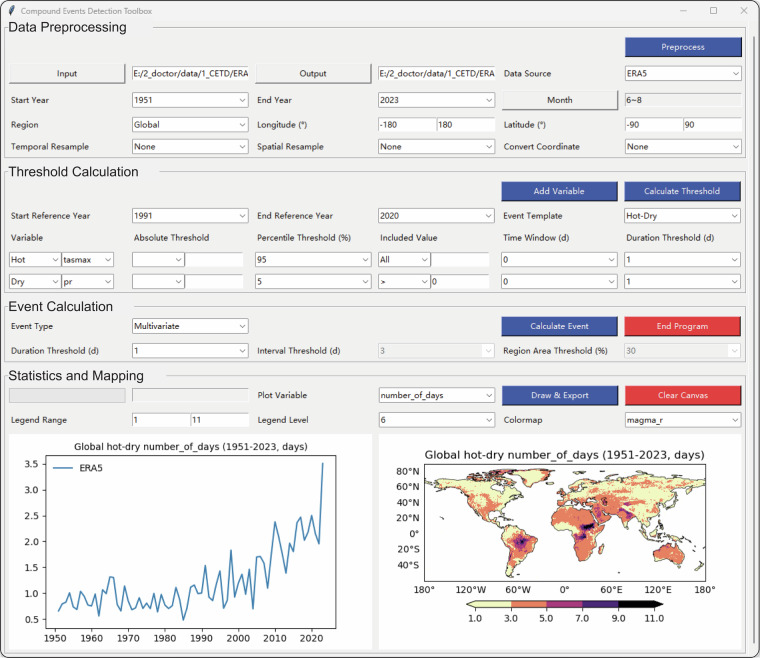
Graphical user interface of CETD.

Following the method introduced above, CETD further provides an integrated, interactive, and extensible Python-based toolbox for detecting and visualizing CEs. Figure 3 shows the graphical user interface of CETD, which includes four modules. (i) The Data Preprocessing module is used to set input/output file paths, data sources, time periods, and regions. It also provides resampling and coordinate conversion functions to standardize the resolution and coordinate system across different input data. (ii) The Threshold Calculation module provides absolute, percentile, and combined thresholds for calculating the threshold files required to detect individual events. (iii) The Event Calculation module provides functions to detect multivariate, sequential, and concurrent events. (iv) The Statistics and Mapping module can display time series and multi-year averages of CE characteristics.

Tables 3 to 6 provide a comprehensive list of adjustable parameters for the four modules of CETD. The toolbox has been optimized in various ways for ease of use. (i) The “Month” button can select the season, consecutive months, or non-consecutive months of interest through the sub-window to meet different usage needs. For example, to detect summer (December-February) hot-dry events in the Southern Hemisphere. (ii) The “Event Template” widget allows selecting the CE of interest and automatically sets the parameters of each variable to commonly used values, reducing manual operations for the user. (iii) The ‘Time Window’ widget allows for calculating thresholds on both seasonal and daily scales. When set to 0, the threshold is calculated over the selected season. When set to n (where n ≥ 1), the threshold is calculated based on a time window consisting of n days before and after the selected date. (iv) The “Draw & Export” button facilitates outputting charts and table files to aid the user’s subsequent analysis.

**Table 3:** Adjustable parameters of the CETD Data Preprocessing module.

Module	Parameter	Description
Data Preprocessing	Input / Output	File paths for input data (e.g., daily temperature, precipitation, wind speed in NetCDF format) and output data (CE records in NetCDF format).
	Data Source	The name of the data source, such as CRU-JRA, ERA5, GLDAS, and CMIP6 GCM.
	Start Year / End Year	The year range for the CE to be identified.
	Month	The month/season of the CE to be identified for each year.
	Region	The region where the CE is to be identified. It can be selected from a list of countries/regions or entered manually.
	Longitude (°) / Latitude (°)	When “Region” is entered manually, the region is a box determined by longitude and latitude.
	Temporal Resample	Resample the input data to daily data.
	Spatial Resample	Resample the input data to the selected spatial resolution.
	Convert Coordinate	Adjust the latitude and longitude of the input data to ascending order.

**Table 4:** Adjustable parameters of the CETD Threshold Calculation module.

Module	Parameter	Description
Threshold Calculation	Event Template	CEs to be identified, such as hot-dry, hot-wet, and hot-dry-stagnation. Once selected, the following 6 threshold-related parameters will be set to common values at the same time.
	Variable	Climate variables used to identify extreme events, such as daily maximum temperature (tasmax), daily total precipitation (pr).
	Absolute Threshold	Grid points that meet this condition will be identified as extreme events, such as temperature “ > 35” °C.
	Start Reference Year / End Reference Year / Percentile Threshold (%) / Included Value / Time Window (days)	Grid points that meet this condition will be identified as extreme events. An example is: If Start Reference Year = 1991, End Reference Year = 2020, Variable = ‘tasmax’, Percentile Threshold (%) = 95, Included Value = ‘ > 0’, Time Window (days) = 7, then this condition is: the 95th percentile of the tasmax set, which consists of the tasmax greater than 0 for 7 days before and after the date from 1991 to 2020.

**Table 5:** Adjustable parameters of the CETD Event Calculation module.

Module	Parameter	Description
Event Calculation	Event Type	The types of CEs to be identified, including multivariate, sequential, and concurrent events.
	Duration Threshold (days)	CEs that persist continuously beyond this threshold will be identified.
	Interval Threshold (days)	When the time interval between two extreme events at a location is shorter than this threshold, they are identified as sequential events.
	Region Area Threshold (%)	When determining whether concurrent events have occurred between the selected region and the remaining grid points, it is necessary to first assess whether extreme events have taken place in the selected region. If the proportion of the area affected by extreme events exceeds this threshold, the region will be considered as having experienced an extreme event.

**Table 6:** Adjustable parameters of the CETD Statistics and Mapping module.

Module	Parameter	Description
Statistics and Mapping	Plot Variable	Selected event characteristics will be displayed on graphs and maps, with options including extent, number_of_events, number_of_days, duration, severity, start date, and end date.
	Legend Range / Legend Level / Colormap	Customize the value range, levels and colormap of the spatial map colorbar respectively.

The limitations of CETD mainly fall into three aspects. Firstly, as mentioned earlier, studies have shown that even when the variables involved in CEs do not exhibit extreme values, they can still have a disproportionate impact^6,7^. For instance, moderate flooding during above-average tides can pose substantial risks to coastal areas^44^. However, CETD assumes that the variables involved in CEs are extreme values, which may introduce bias in the identification of CEs. Secondly, CETD can identify CE involving climate variables, while generalized CE may involve non-climate variables such as floods, crop failures, landslides, etc^18^. Thirdly, CETD does not include a parameter to filter the areal extent of CEs or to cluster events that are closer than a certain threshold distance. This may not fully meet users' needs to focus specifically on events with a large extent or to consider only those events that are spatially compounding and separated by a minimum distance. Our further effort is to create an online version of CETD with prepared data sources and diverse analysis and visualization methods, as well as a function to rank CEs under different thresholds^45,46^. Future work could also expand to include additional datasets (e.g., weather forecasting models), climate variables (e.g., cold extremes), and secondary non-climate variables (e.g., crop yields, or solid earth hazards). The database could also be used by sectoral experts (e.g., hydrologists or water managers) aiming to better understand how climate may have impacted their sector, and how climate change will impact the future viability of resilience and greenhouse gas mitigation solutions. We believe that CETD will play an important role for climate scientists, practitioners, and the public to recognize and respond to CEs.

## Data Records

CETD provides daily climate data sourced from ERA5, CRU-JRA, GLDAS^23–26^, which serve as input for the toolbox to generate percentile thresholds and subsequently identify CEs. ERA5 daily data are synthesized from hourly data at both single and 500hPa pressure levels, while data from the other sources are retrieved and pre-processed from their daily records. Variable names across different sources have been standardized, with daily maximum temperature, total precipitation, mean surface wind speed, and mean wind speed at 500 hPa being denoted as tasmax, pr, sfcWind, and preWind500, respectively. Additionally, CETD provides yearly CE characteristics data derived from ERA5 and six CMIP6 GCMs^27^, encompassing metrics such as frequency, duration, and intensity of hot-dry, hot-wet, hot-stagnation, wet-windy, hot-dry-stagnation, hot-dry-windy, and hot-wet-stagnation events. These datasets are accessible via figshare^47^. A list of available variables and time periods is presented in Table 7. We note that these datasets represent a subset of the possible outputs from CETD. Both the data sources, time periods, variables, and CE types can be expanded by leveraging CETD’s multiple adjustable parameters.

**Table 7:** Available variables and time periods for CETD dataset.

Data source	Time period	Daily climate data variable	Yearly CE attribute data	
			CE	Characteristic
ERA5	1951–2022	tasmax^a,b^ pr^a, b^ sfcWind^a, b^ preWind500^a^	hot-dry^a,c^ hot-wet^a, c^ hot-stagnation^a^ wet-windy^a, c^ hot-dry-stagnation^a^ hot-dry-windy^a, c^ hot-wet-stagnation^a^	Number of events Number of days Maximum duration Minimum duration Average duration Average severity Peak severity Start date End date
CRU-JRA	1951–2021			
GLDAS	1951–2014			
ACCESS-CM2 CMCC-ESM2 MIROC6 MPI-ESM1–2-LR MRI-ESM2-0 NorESM2-MM	1951–2014			
	2015–2100			

^a^Available for ERA5 data.

^b^Available for CRU-JRA and GLDAS data.

^c^Available for six CMIP6 GCMs data.

## Technical Validation

To demonstrate CETD’s capabilities, we present statistics on CEs from several recent high-impact events, including the European heatwave of 2022, the Australian wildfires of 2019, the Pakistan floods of 2022, and the Texas heatwave of 2023, all of which resulted in severe social and ecological impacts. The data and maps presented in this section are directly from CETD’s Statistics and Mapping module and have only been merged and labelled without further editing or post-processing.

### Multivariate events during the European heatwave of 2022

Over the past few decades, Europe has experienced several record-breaking heatwaves^48^, making it one of the global heatwave hotspots^49^. In the summer of 2022, the average temperature in Europe was 0.4 °C higher than 2021, becoming the hottest season on record, with severe heatwaves engulfing the entire continent^50^. At the same time, large parts of Western Europe experienced reduced precipitation and exceptional dryness, resulting in one of the worst droughts in recent history^51^. An estimated 61,672 people died from summer heatwaves in Europe in 2022^52^, while reduced river flows and hydrological droughts impacted public water supplies, hydropower, and commercial inland water transport^53^.

**Fig. 4:**
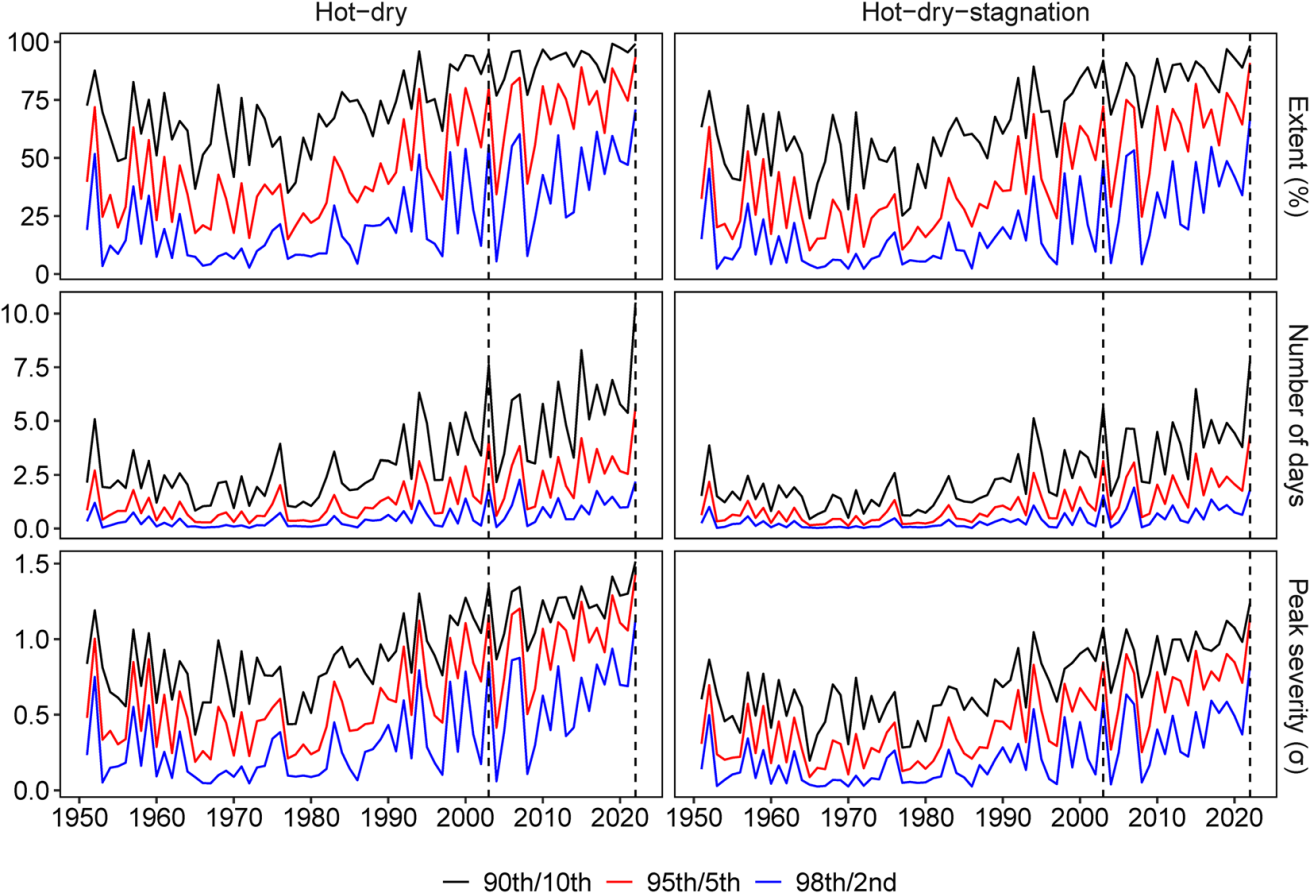
The extent (first row), number of days (second row), and peak severity (third row) of hot-dry events (first column) and hot-dry-stagnation events (second column) in Europe during the summers from 1951 to 2022 based on three threshold combinations: 90th/10th (black line), 95th/5th (red line), and 98th/2nd (blue line). The definitions of extent, number of days, and peak severity of events are detailed in Table 2. The same definitions apply to the figures below.

Figure 4 illustrates the time series of hot-dry and hot-dry-stagnation events in Europe (10°W-30°E, 36°N-59°N) during the summers (June, July, and August) from 1951 to 2022 under different threshold combinations. The red line depicts the results based on Table 1, which utilizes a 95th/5th percentile threshold to define hot and dry events, respectively. The black and blue lines employ a similar definition strategy while using a 90th/10th and 98th/2nd percentile, respectively. Overall, results based on different threshold combinations consistently reveal an upward trend in the extent, number of days, and peak severity of hot-dry and hot-dry-stagnation events. All lines demonstrate the extremeness of these two CEs in the summers of 2003 and 2022, which experienced severe heatwaves. Based on the results derived from the 95th/5th percentile threshold, it is observed that more than 60% of Europe’s land areas have experienced at least one hot-dry and hot-dry-stagnation event every summer since the beginning of the 21st century. During the unprecedented heatwave of summer 2022, Europe witnessed record-breaking hot-dry and hot-dry-stagnation events. The average total number of hot-dry and hot-dry-stagnation days reached 6 and 4 days, respectively, which were twice as high as the average level. The peak severity was also twice the average, with the maximum mean anomalies of the climate variables involved in the two types of CEs reaching 1.5 and 1.2 standard deviations (σ), respectively. The extent, number of days, and peak severity of hot-dry and hot-dry-stagnation events in 2022 surpassed those of all previous years since 1951, underscoring the extremeness of the European climate in the summer of 2022.

**Fig. 5:**
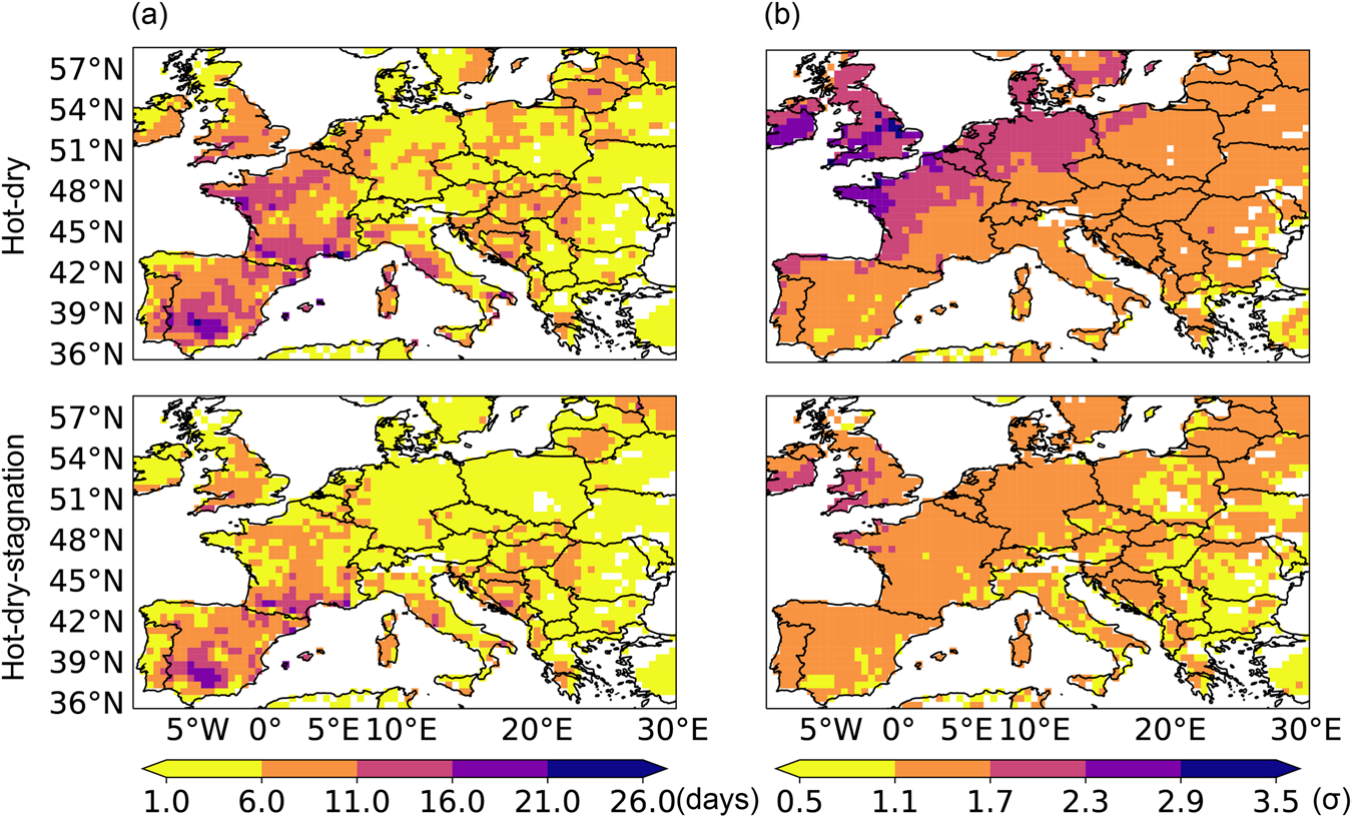
Number of days (**a**) and peak severity (**b**) of dry-hot (first row) and dry-hot stagnation events (second row) during the summer of 2022. These results are calculated based on the 95th/5th percentile threshold.

Figure 5 depicts the spatial distribution of hot-dry and hot-dry-stagnation events in Europe during the summer of 2022 based on the 95th/5th percentile thresholds. Hot-dry events swept across Europe, with some regions in Spain, France, and Italy experiencing more than 16 days of hot-dry conditions. Northern Europe encountered more severe hot-dry events compared to Southern Europe, with peak severity surpassing 1.7σ in the UK, Ireland, and Nordic countries. Similarly, hot-dry-stagnation events in Spain and France were very severe, with certain areas experiencing more than 11 days of hot-dry-stagnation conditions. Considering that stagnation may amplify the heat risk for individuals, for instance, by being associated with clear skies and diminished air quality^54^, hot-dry-stagnation events warrant increased attention.

### Multivariate events during the Australian wildfires of 2019

Between July 2019 and January 2020, Australia experienced record-breaking high temperatures and drought, leading to severe wildfires in the region, affecting millions of hectares of land^55^. It is estimated that 33 people died as a direct result of the wildfires, with 429 premature deaths attributed to smoke exposure, carrying a health burden equivalent to A$1.95 billion^56^. Over 3,000 homes were destroyed^57,58^, and approximately 715 million tons of carbon dioxide were released into the atmosphere, equivalent to 1.5 times the country’s emissions for the year 2022^59–61^.

**Fig. 6:**
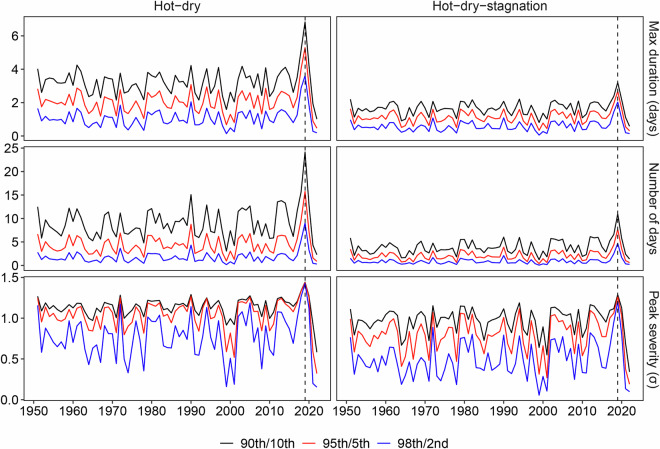
Maximum duration (first row), number of days (second row), and peak severity (third row) of hot-dry events (first column) and hot-dry-stagnation events (second column) in Australia in the second half of the year from 1951 to 2022 based on three threshold combinations: 90th/10th (black line), 95th/5th (red line), and 98th/2nd (blue line).

Figure 6 illustrates the time series of hot-dry and hot-dry-stagnation events in Australia in the second half of the year (July to December) from 1951 to 2022. The results, derived from different threshold combinations, consistently captured peak values for both two CEs in 2019 across all three measured characteristics. Based on the 95th/5th percentile threshold, the maximum duration, number of days, and peak severity of hot-dry and hot-dry-stagnation events in 2019 were observed to be 2-3 times higher than their respective average values. This indicates that the extremely hot, dry, and stagnant weather conditions in 2019 may have significantly contributed to the severe wildfires experienced during that year.

**Fig. 7:**
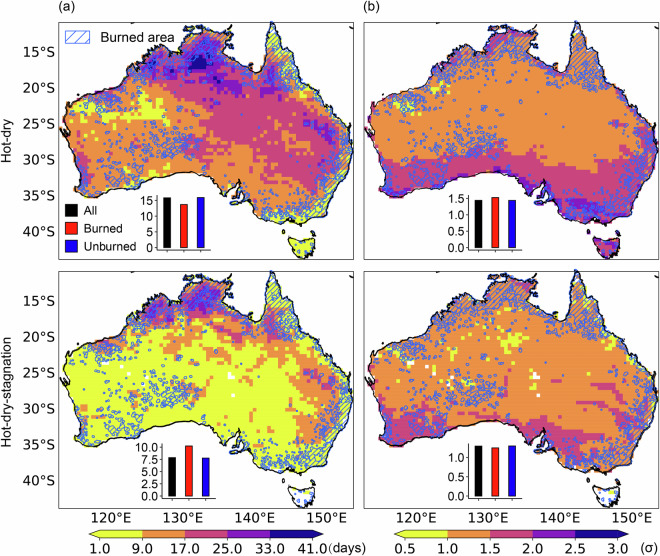
Number of days (**a**) and peak severity (**b**) of dry-hot (first row) and hot-dry-stagnation (second row) events in the second half of 2019. These results are calculated based on the 95th/5th percentile threshold. The blue grid represents the location of burned area. The histograms display the averages of the entire country (black), the burned area (red), and the unburned area (blue). The burned area data is derived from the National Fire Indicator Range Summary dataset and buffered to avoid fragmentation^76^.

Figure 7 illustrates the key statistics of hot-dry and hot-dry-stagnation events in Australia during the second half of 2019 based on the 95th/5th percentile thresholds, in combination with burned area. Hot-dry events were detected across the entire country, with number of days exceeding 9 and peak severity surpassing 1σ in most regions. Despite various non-climatic factors such as vegetation distribution, lightning, anthropogenic ignition, and land-use policies potentially influencing wildfire occurrence^62^, the number of days of hot-dry and hot-dry-stagnation events, along with burned areas, exhibited notable spatial consistency, particularly in the North Coastal and Southwestern regions. Histograms further illustrate the average number of days and peak severity for the entire country (black), burned areas (red), and unburned areas (blue), emphasizing that the number of days of hot-dry-stagnation events in burned areas exceeded that in unburned areas by 2.47 days. Considering that stagnation may elevate air pollutant concentrations and reduce visibility^63,64^, hot-dry-stagnation events may pose greater challenges for wildfire management as well.

### Sequential events during the Pakistan floods of 2022

In the summer of 2022, intense rainfall led to severe flooding in Pakistan. Approximately one-third of the country was submerged, resulting in around 2,000 deaths, and displacing 32 million people^65,66^, with estimated economic losses reaching $15.2 billion^67^. Prior to the summer floods, Pakistan experienced devastating heatwaves during the spring. Research indicates that the heatwaves and associated glacier melting exacerbated the impact of rainfall on these catastrophic floods. The rise in surface temperatures during heatwaves may have intensified low-pressure systems, facilitating the movement and expansion of monsoon lows along the Arabian Sea coast, thus exacerbating heavy precipitation. Meanwhile, accelerated glacier melting in northern Pakistan during heatwaves increased the risk of flooding in mountainous regions^68–70^.

**Fig. 8:**
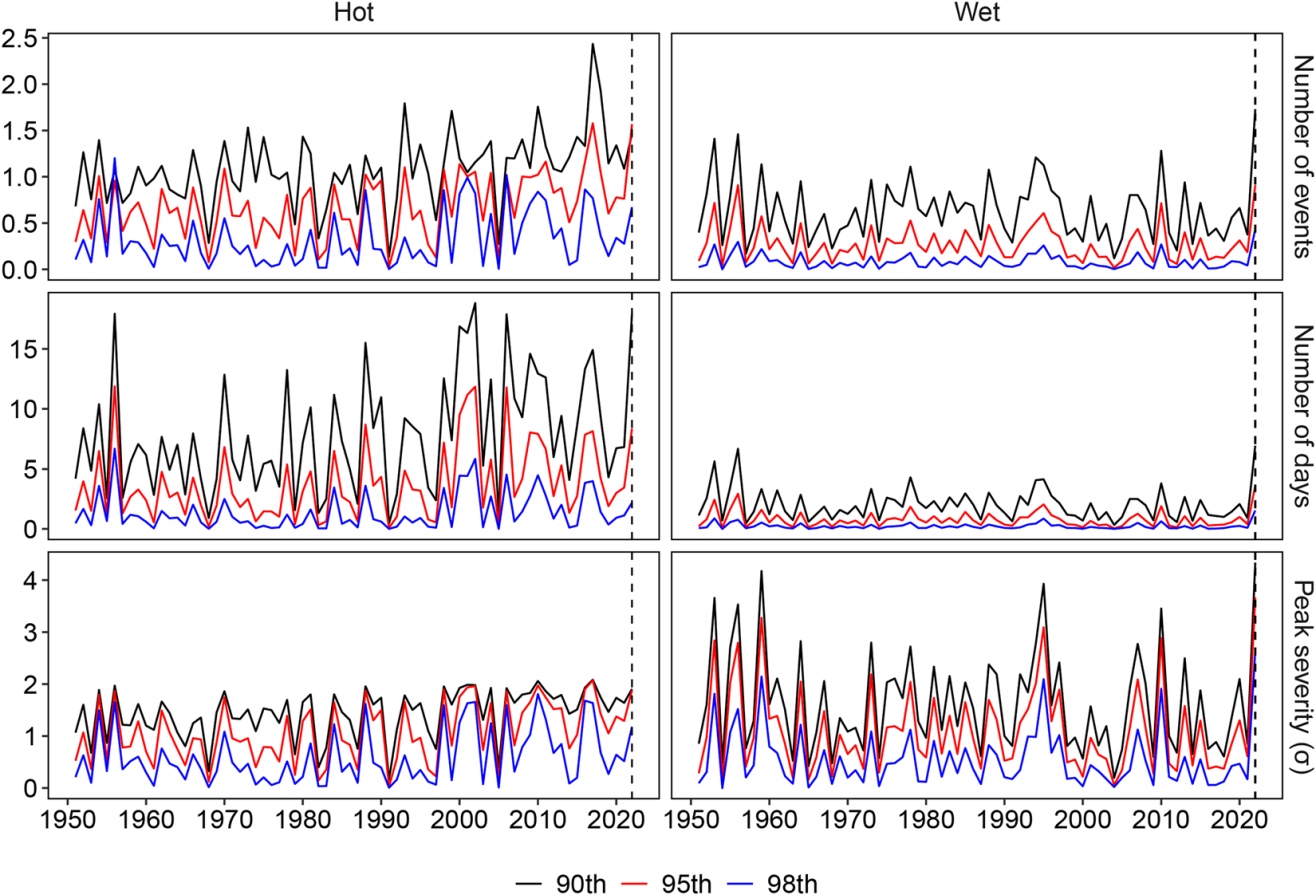
Number of events (first row), number of days (second row), and peak severity (third row) of spring sequential hot events (first column) and summer sequential wet events (second column) in Pakistan from 1951 to 2022 based on three threshold combinations: 90th/10th (black line), 95th/5th (red line), and 98th/2nd (blue line).

Figure 8 illustrates the time series of spring (March, April, and May) sequential hot events and summer (June, July, and August) sequential wet events in Pakistan from 1951 to 2022. Sequential hot (wet) events are defined as the time interval between individual hot (wet) events at a location being less than 3 days. For instance, if the second hot event occurs within 3 days after the first one, the days between the start date of the first event and the end date of the second event will be identified as a sequential hot event. Over the past two decades, the number of events, number of days, and peak severity of sequential hot events have frequently reached maximum values, indicating an increasing trend of extreme spring heat in Pakistan. In 2022, all three characteristics of sequential hot events remained at notably high levels. Similarly, for sequential wet events, the three measured characteristics also peaked in 2022. These CE findings are consistent with the broader conclusion that during the spring and summer of 2022, Pakistan experienced severe extreme heat and extreme precipitation, and the CE may have been significant contributors to severe flooding.

**Fig. 9:**
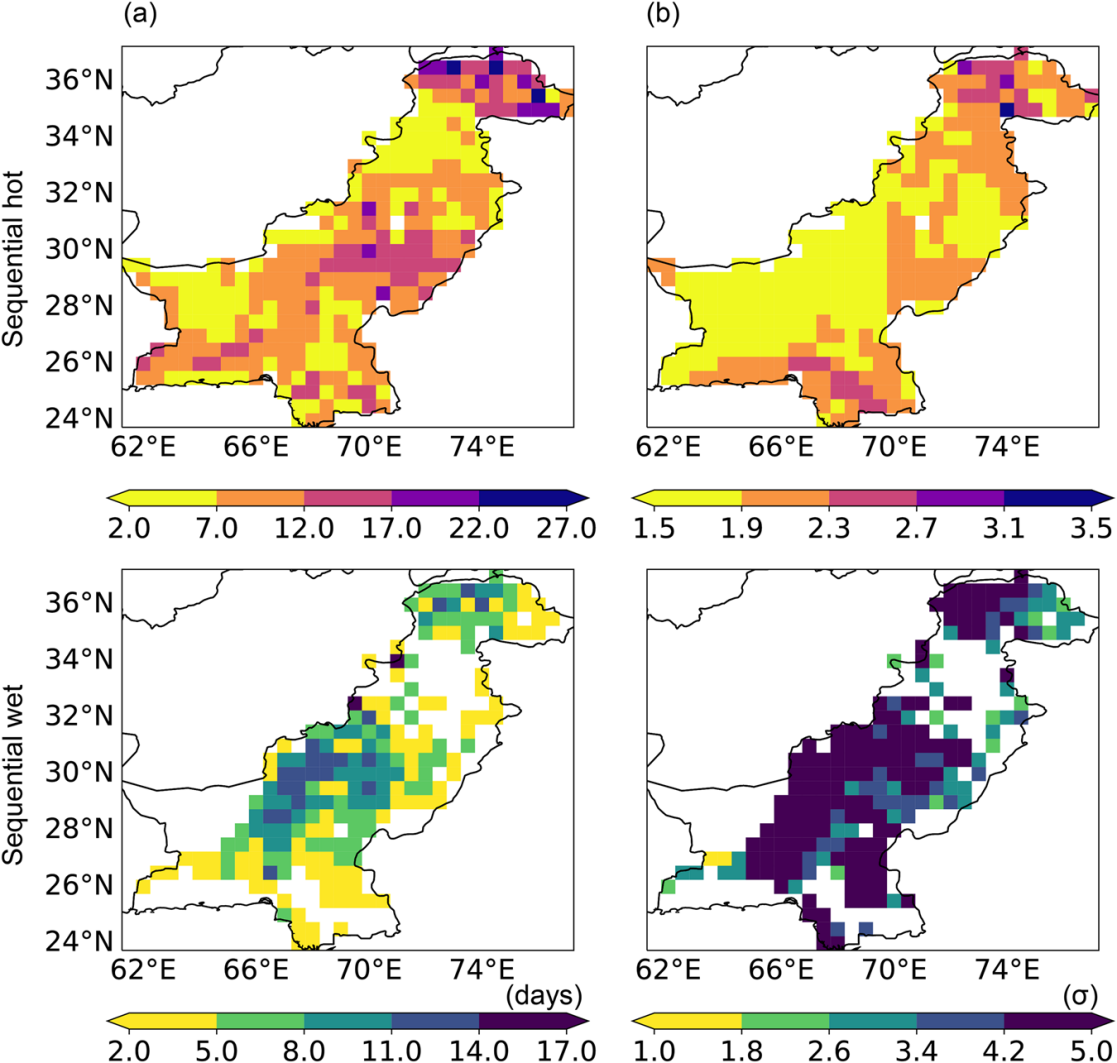
Number of days (**a**) and peak severity (**b**) of spring sequential hot (first row) and summer sequential wet (second row) events in 2022. These results are calculated based on the 95th/5th percentile threshold.

Figure 9 depicts the spatial distribution of spring sequential hot events and summer sequential wet events in Pakistan in 2022 based on the 95th/5th percentile thresholds. In 2022, the number of days of sequential hot events in most parts of Pakistan exceeded 12 days, and the peak severity exceeded 1.5σ, with northern Pakistan being the hotspot. For sequential wet events, these two characteristics reached 5 days and 3.4σ in most areas, with the hotspot shifting to southern Pakistan. This suggests that melting glaciers caused by spring extreme temperatures in northern Pakistan and summer extreme precipitation in the south may have combined to cause the severe flooding in Pakistan in 2022 that largely manifested as fluvial flooding.

### Concurrent events during the Texas heatwave of 2023

In summer 2023, multiple regions in the Northern Hemisphere experienced severe heatwaves, including the Southwest United States, Mexico, Southern Europe, and China^71^. Texas is one of the regions that was most affected by heatwaves in the United States, with 2023 being the state’s hottest year on record^72^. More than 300 people died from heatwaves, the highest since 1989^73^.

**Fig. 10:**
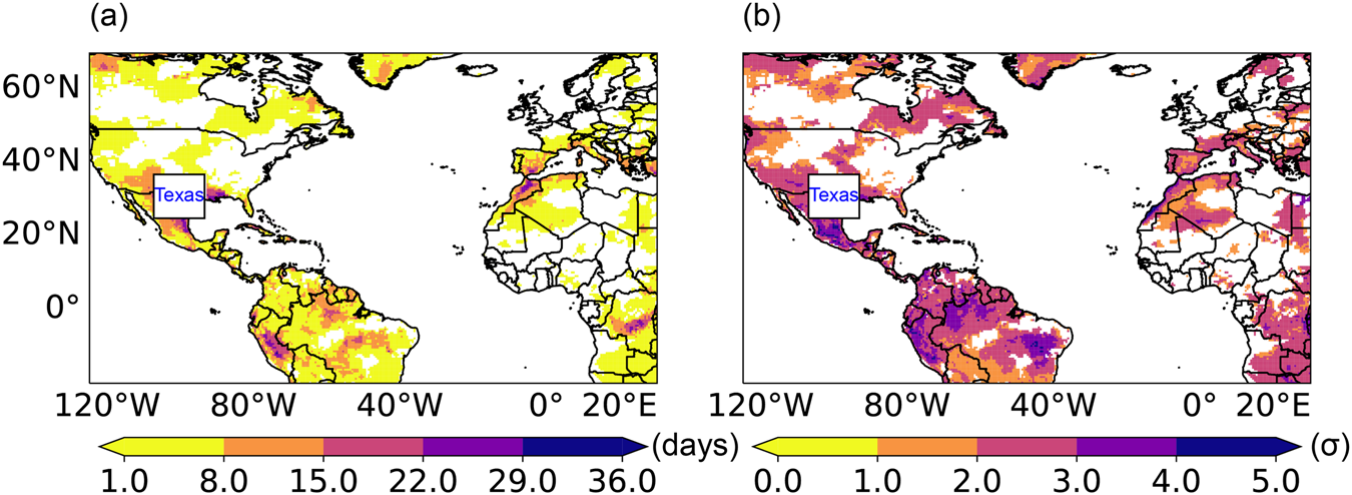
The spatial distribution of concurrent heatwaves in Texas during the summer of 2023: (**a**) the number of days when each grid point (outside the box) and Texas (the box) experience heatwaves concurrently; (**b**) the peak severity of heatwaves during these days.

Figure 10 presents the spatial distribution of concurrent heatwaves in Texas (107°W-93°W, 25°N-37°N) during the summer (June, July, and August) of 2023. Figure 10a displays the number of days when each grid point (outside the box) and Texas (the box) experience heatwaves concurrently, while Fig. 10b illustrates the peak severity of heatwaves during these days. Here, Texas is experiencing a heatwave if over 30% of its area encounters hot events for more than 3 consecutive days, and “concurrently” refers to two locations experiencing heatwaves on the same day. According to Fig. 10a, most regions experienced heatwaves concurrently within Texas, indicating that Texas encountered a prolonged heatwave during the summer of 2023. The southwestern United States and Mexico, adjacent to Texas, experienced over 15 days of heatwave conditions, further highlighting the scale and duration of the event. Northern South America, southern Europe, and eastern Africa also recorded more than 8 heatwave days concurrently with Texas, pointing to the influence of global warming and climate dynamics on the evolution of heatwaves. The peak severity of heatwaves in these concurrent regions is typically high, with southern Europe reaching 2σ, while the southwestern United States and Mexico, Northern South America, and eastern Africa reached 3σ.

### Global statistics of multivariate CEs

**Fig. 11:**
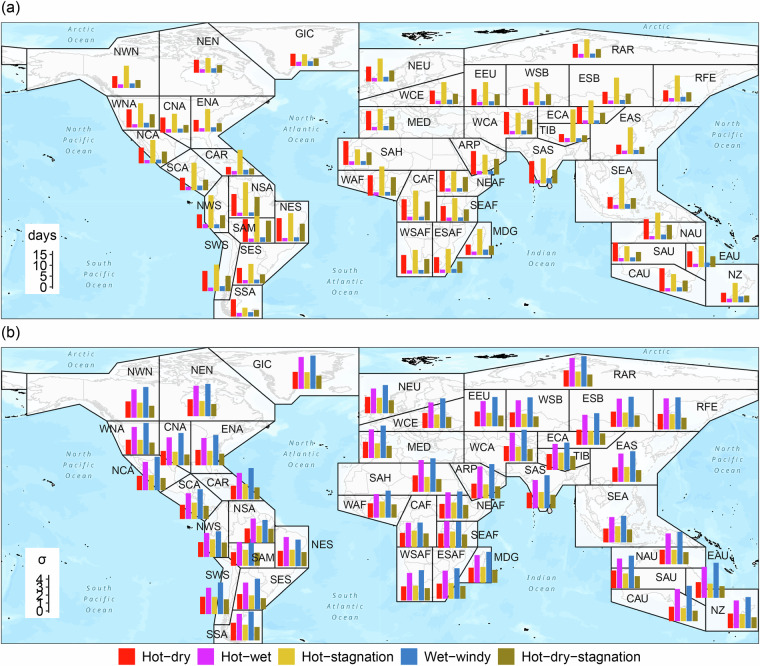
Average number of days (**a**) and average peak severity (**b**) of hot-dry, hot-wet, hot-stagnation, wet-windy, and hot-dry-stagnation events for each SREX region from 1951 to 2022. These multivariate events were detected based on the 95th/5th percentile threshold and ERA5 data.

Figure 11 illustrates the average number of days and average peak severity of 5 multivariate events for each SREX region from 1951 to 2022 based on the 95th/5th percentile threshold and ERA5 data. The SREX zoning scheme comprises 46 land areas and 15 marine areas^74^. The results suggest that multiple CEs may have occurred in all subregions, indicating the potential threat posed by various CEs. It is notable that hot-related CEs, including hot-dry, hot-stagnation, and hot-dry-stagnation events, often exhibit longer durations and relatively lower severity in most subregions, while wet-related events, including hot-wet and wet-windy events, typically have shorter durations and higher severity. This suggests that hot-related CEs may occur frequently but often do not directly lead to severe consequences. For instance, the occurrence of drought typically necessitates prolonged high temperatures and low rainfall. On the other hand, wet-related events occur less frequently, but once they occur, they can result in catastrophic consequences, such as typhoons.

The prevalence of dominant CEs exhibits a distinct pattern in latitudinal distribution. Moving towards higher latitudes, the average number of days of hot-related events tends to decrease, while wet-related events tend to increase. Conversely, for average peak severity, CEs generally display an increasing trend when moving towards higher latitudes, which may be attributed to more pronounced climate changes in these regions^75^. Notably, three-variable CEs, such as hot-dry-stagnation events, occur with high frequency in most tropical and subtropical regions. These events deserve our focus due to their potential for significant social and ecological impacts.

## Usage Notes

To use the toolbox, users should first download and unzip the “CETD_v3.1.zip” file, and then run “CETD_v3.1.exe.” Alternatively, users can directly download and run “CETD_v3.1.py,” though this method requires the installation of dependent packages. It is recommended that users refer to Tables 3 through 6 to familiarize themselves with the toolbox parameters.

For input data, users can utilize ERA5, CRU-JRA, and GLDAS daily data provided alongside the toolbox as input data, or they may generate daily data from other sources independently. The input data must meet certain basic requirements. First, it may contain multiple variables depending on the definition of the CEs of interest. For example, daily maximum temperature and daily total precipitation are needed to identify hot-dry events in this study. Second, the input data should be organized in yearly NetCDF files, each containing one variable and structured with three dimensions (latitude, longitude, and time). Third, NetCDF files for different variables should share the same dimensions and be stored in a single folder.

Users are encouraged to view the tutorial video and begin working with the provided input data. All mentioned code, tool, data, and tutorial video are available on figshare^47^.

## Supplementary information

Supplementary Information

## Data Availability

The CETD toolbox (CETD_v3.1.exe), source code (CETD_v3.1.py), and tutorial video (CETD_tutorial.mp4) are available on figshare^47^.
