# Supplementary material for: CETD, a global compound events detection and visualisation toolbox and dataset
Source: Sci Data. 2025 Feb 28;12:356. doi: 10.1038/s41597-025-04530-x (PMC11871071; doi:10.1038/s41597-025-04530-x)
Supplement: Supplementary file 1 — Supplementary Information [file 41597_2025_4530_MOESM1_ESM.docx]

### Title

*CETD, a global compound events detection and visualisation toolbox and dataset*

### Authors

Cong Yin^1,2,6^, Mingfang Ting^2,3^, Kai Kornhuber^2,4,5^, Radley M. Horton^2,3^, Yaping Yang^1,7^, Yelin Jiang^2^

**Affiliations**

1. Institute of Geographic Sciences and Natural Resources Research, Chinese Academy of Sciences, Beijing, China

2. Lamont-Doherty Earth Observatory, Columbia University, Palisades, USA

3. Columbia Climate School, Columbia University, New York, USA

4. Climate Analytics, Berlin, Germany

5. German Council on Foreign Relations, Berlin, Germany

6. College of Resources and Environment, University of Chinese Academy of Sciences, Beijing, China

7. Jiangsu Center for Collaborative Innovation in Geographical Information Resource Development and Application, Nanjing, China

corresponding author(s): Yaping Yang ([yangyp@igsnrr.ac.cn](mailto:yangyp@igsnrr.ac.cn)), Cong Yin (cy2722@columbia.edu)

### Comparison with existing studies

To verify that CETD can accurately identify compound events, we compared the identification results of wet-windy events based on CETD with those from Ridder et al ^1^. For this comparison, we employed a consistent approach to identify wet-windy events: daily precipitation greater than the 99th percentile (considering only days with daily precipitation >1 mm) and daily maximum wind speed greater than the 99th percentile (considering only days with daily maximum wind speed >0.5 m/s). The reference period used was 1980-2014. The results, presented as return periods in Figure S1, represent the average interval between two recurring events. Overall, the two sets of results exhibit consistent spatial patterns, with CETD-based results offering higher resolution. The areas where wet-windy events occur and their return periods are largely consistent, with the coastal regions of North America, southwestern Europe, northern Australia, India, and southeastern China all identified as hotspots for wet-windy events.


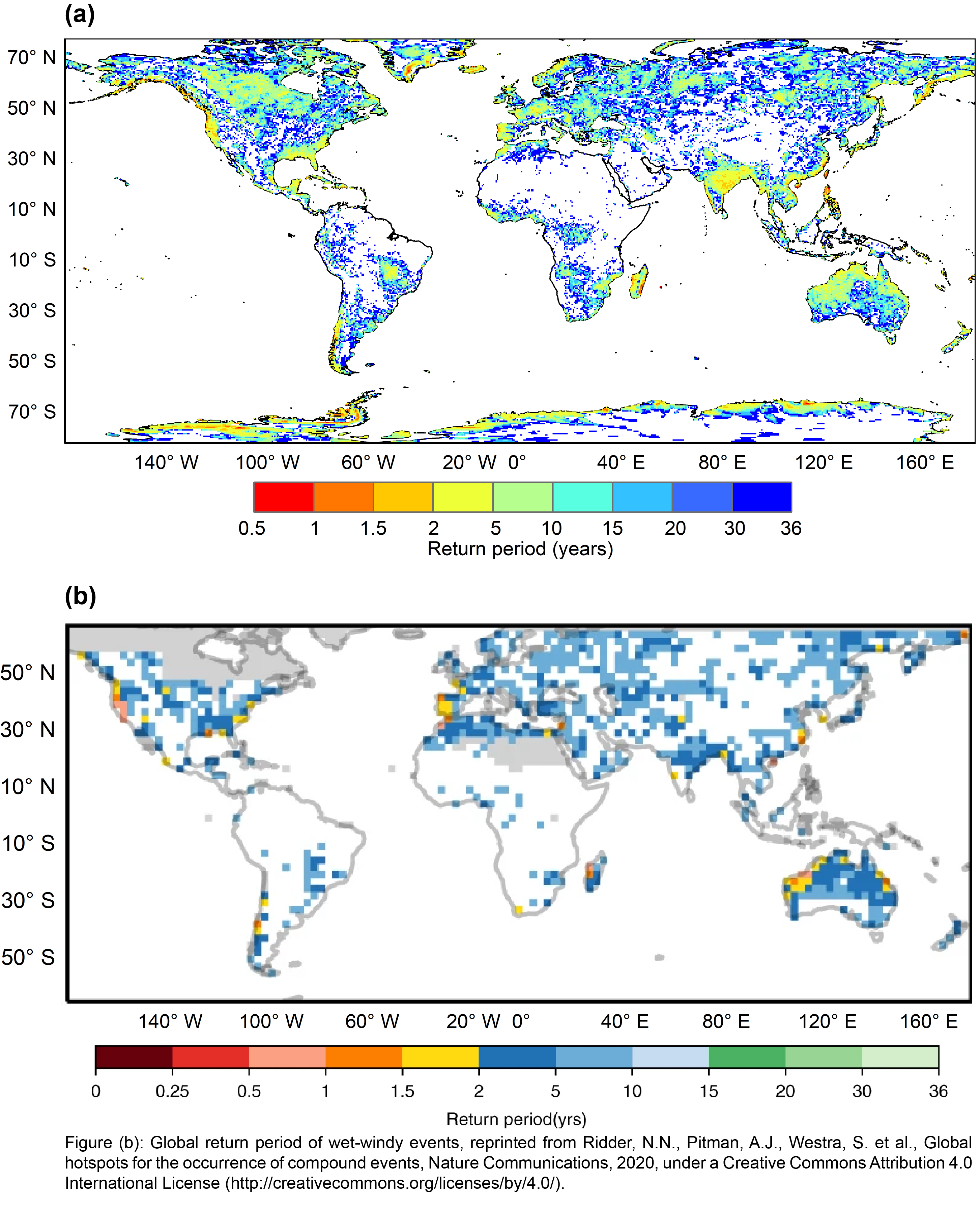


Figure S1. Global return period of wet-windy events produced by (a) CETD and (b) Ridder, Pitman et al., 2020.

### Multivariate events during the European heatwave of 2022

Figures S2 and S3 depict the spatial distribution of hot-dry and hot-dry-stagnation events in Europe during the summer of 2022, based on the 90th/10th and 98th/2nd percentile thresholds, respectively.


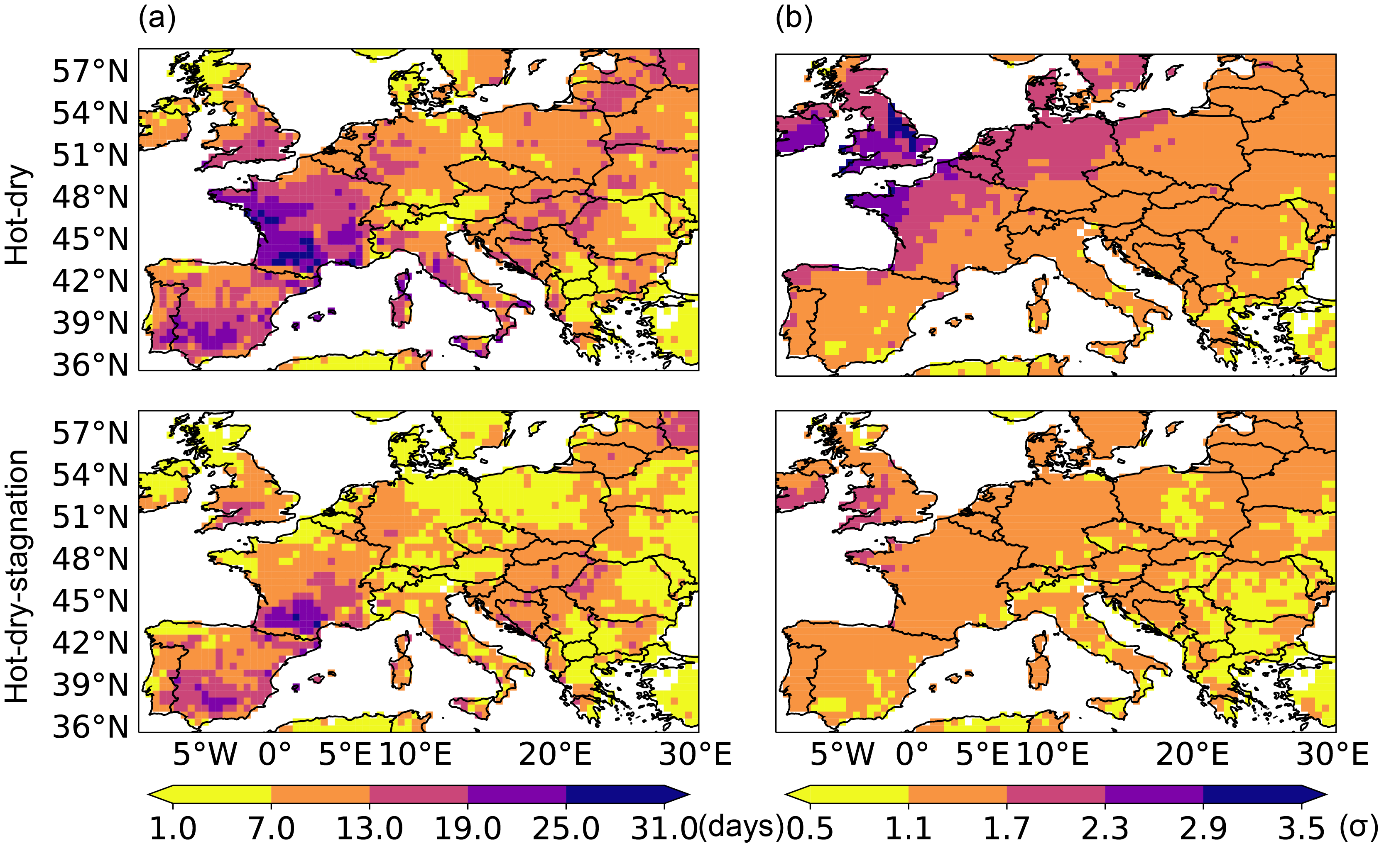


Figure S2. Number of days (a) and peak severity (b) of dry-hot (first row) and dry-hot stagnation events (second row) in Europe during the summer of 2022. These results are calculated based on the 90th/10th percentile threshold.


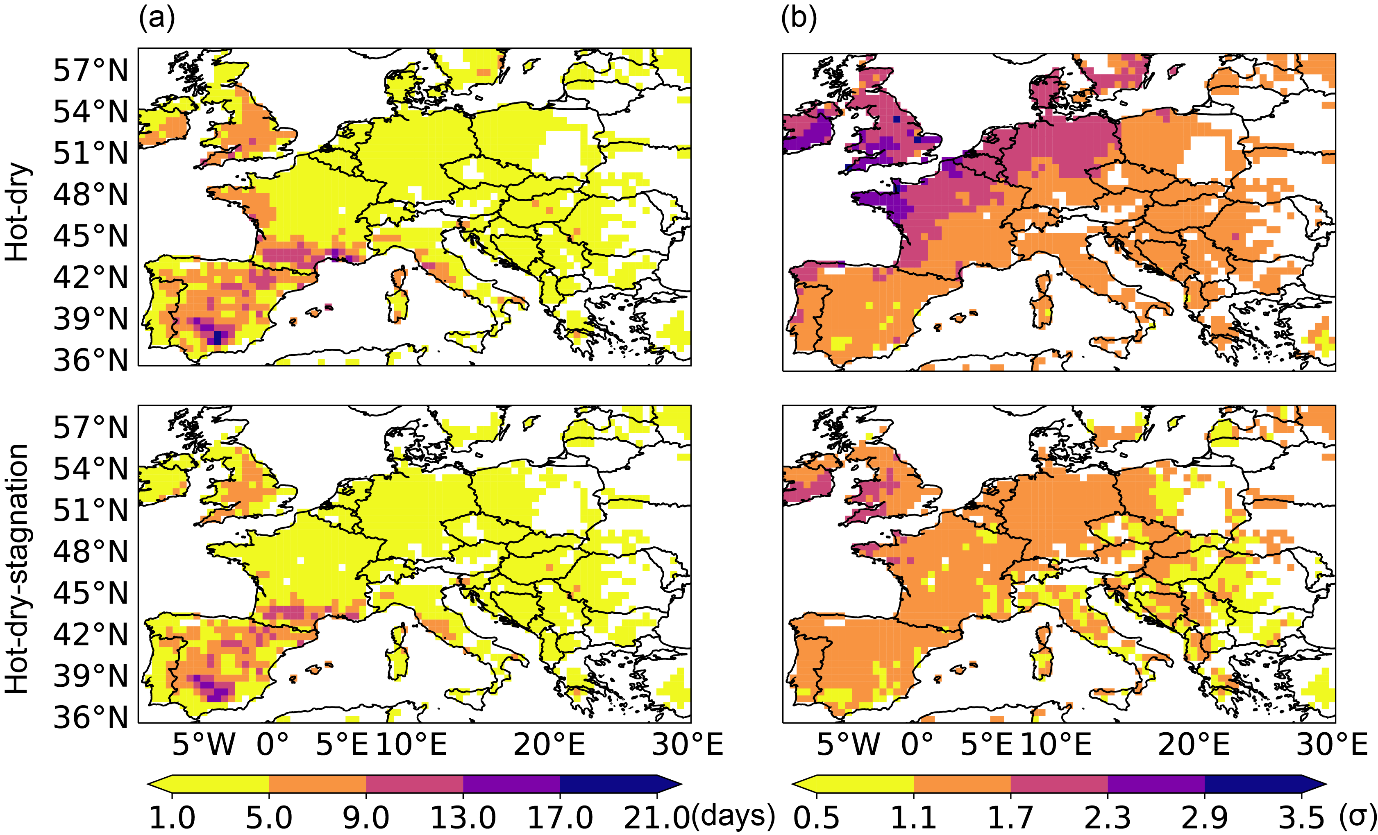


Figure S3. Number of days (a) and peak severity (b) of dry-hot (first row) and dry-hot stagnation events (second row) in Europe during the summer of 2022. These results are calculated based on the 98th/2nd percentile threshold.

**Multivariate events during the Australian wildfires of 2019**

Figures S4 and S5 illustrate the key statistics of hot-dry and hot-dry-stagnation events in Australia during the second half of 2019, based on the 90th/10th and 98th/2nd percentile thresholds, respectively.


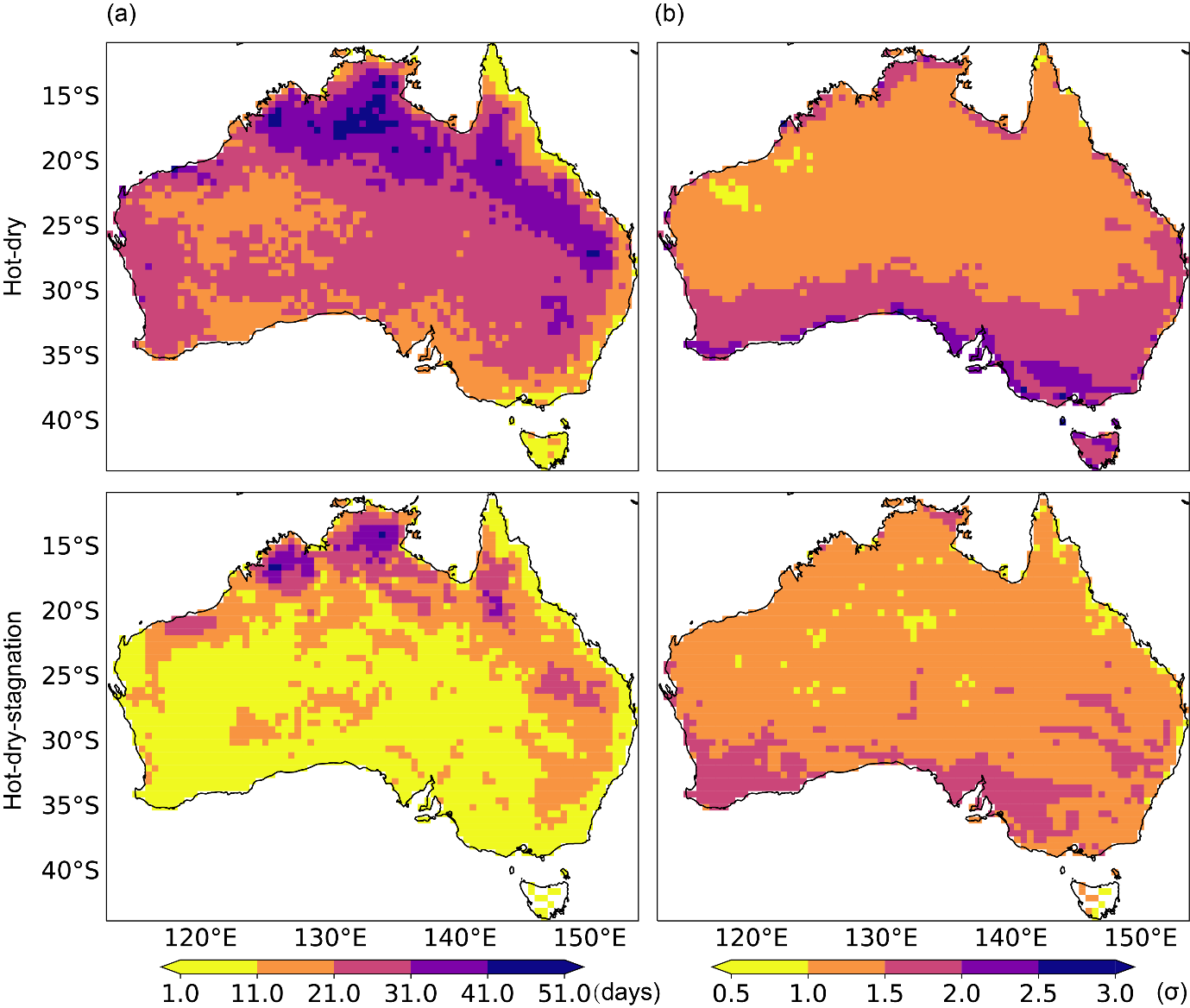


Figure S4. Number of days (a) and peak severity (b) of dry-hot (first row) and hot-dry-stagnation (second row) events in Australia in the second half of 2019. These results are calculated based on the 90th/10th percentile threshold.


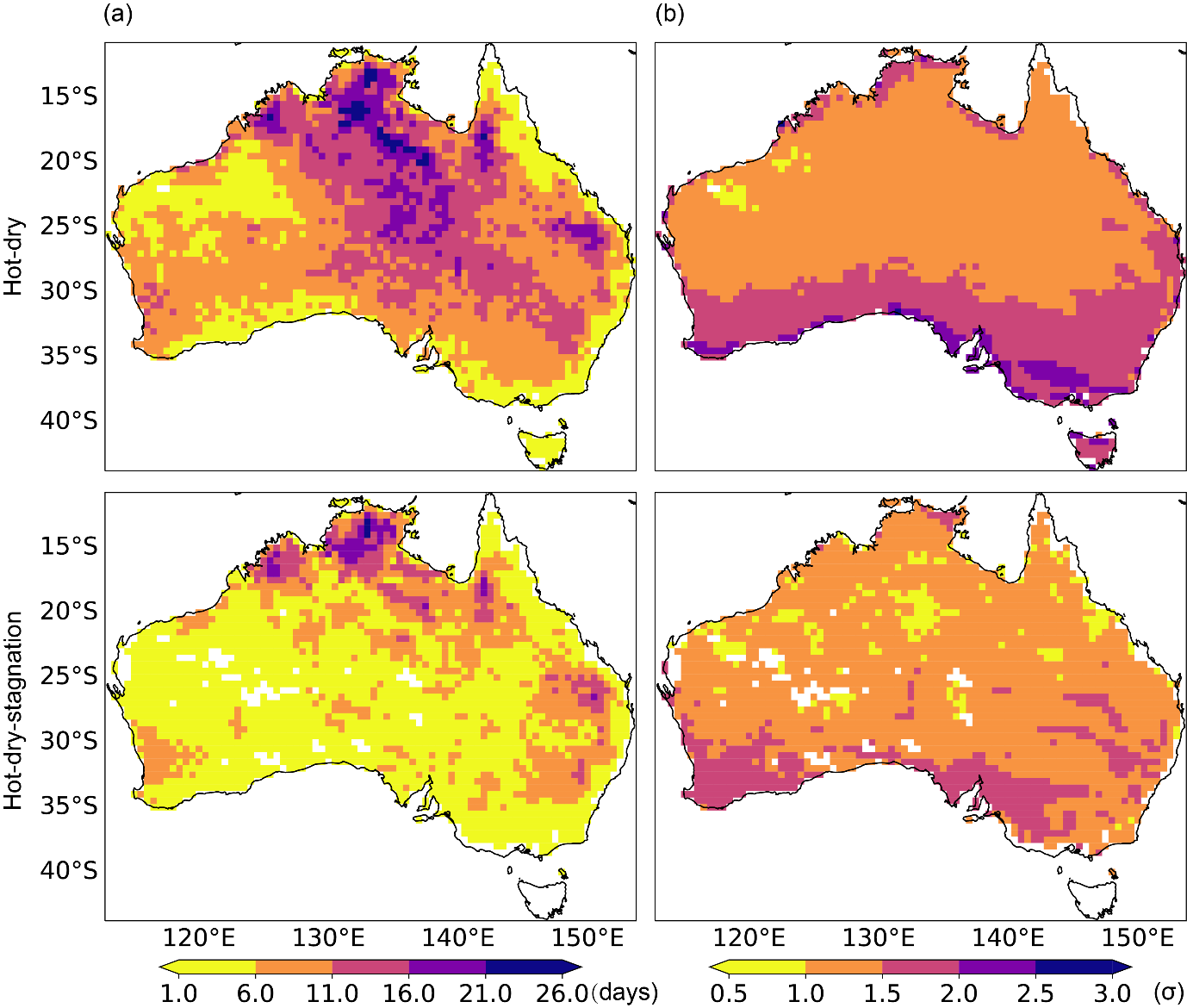


Figure S5. Number of days (a) and peak severity (b) of dry-hot (first row) and hot-dry-stagnation (second row) events in Australia in the second half of 2019. These results are calculated based on the 98th/2nd percentile threshold.

**Sequential events during the Pakistan floods of 2022**

Figures S6 and S7 depict the spatial distribution of spring sequential hot events and summer sequential wet events in Pakistan in 2022, based on the 90th/10th and 98th/2nd percentile thresholds, respectively.


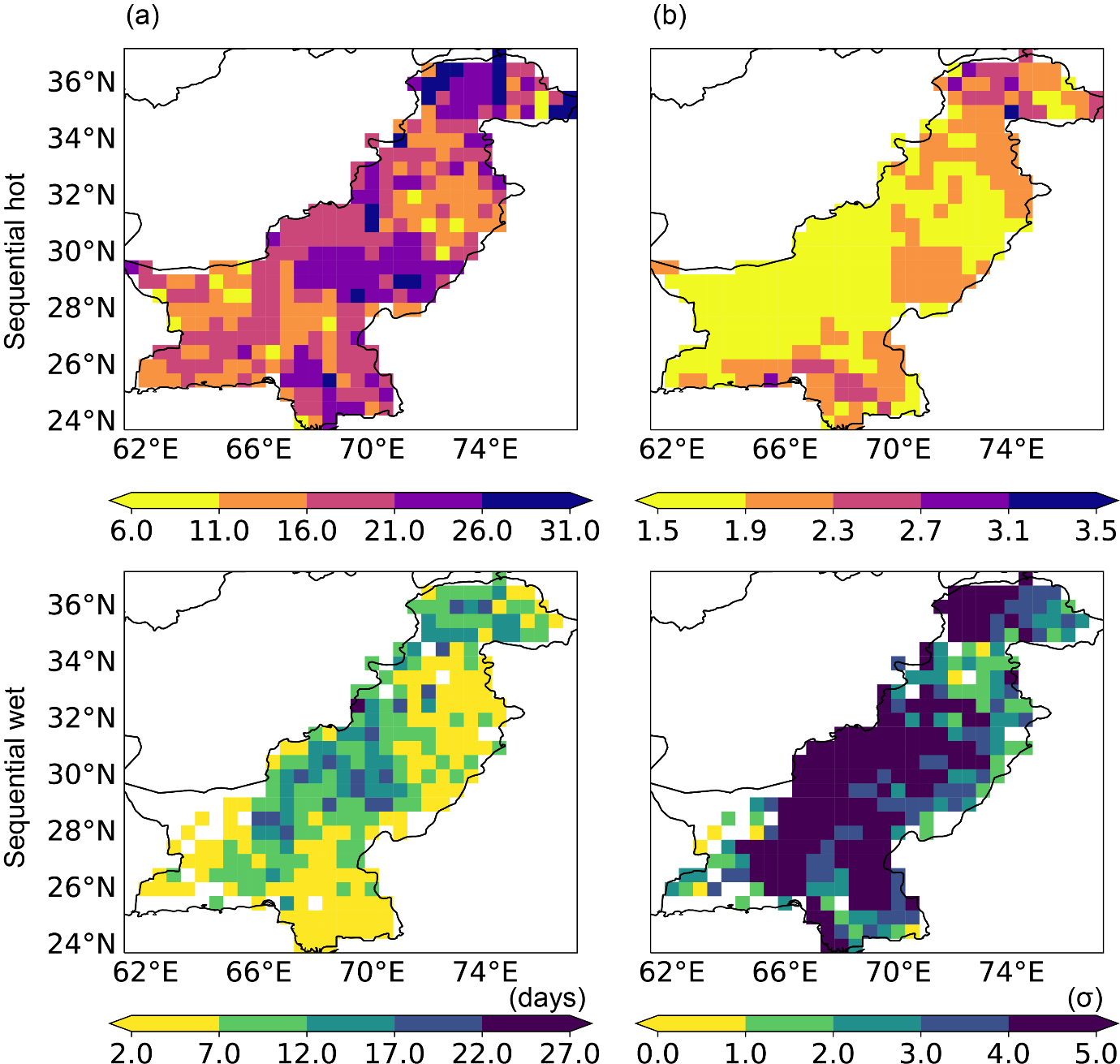


Figure S6. Number of days (a) and peak severity (b) of spring sequential hot (first row) and summer sequential wet (second row) events in Pakistan in 2022. These results are calculated based on the 90th/10th percentile threshold.


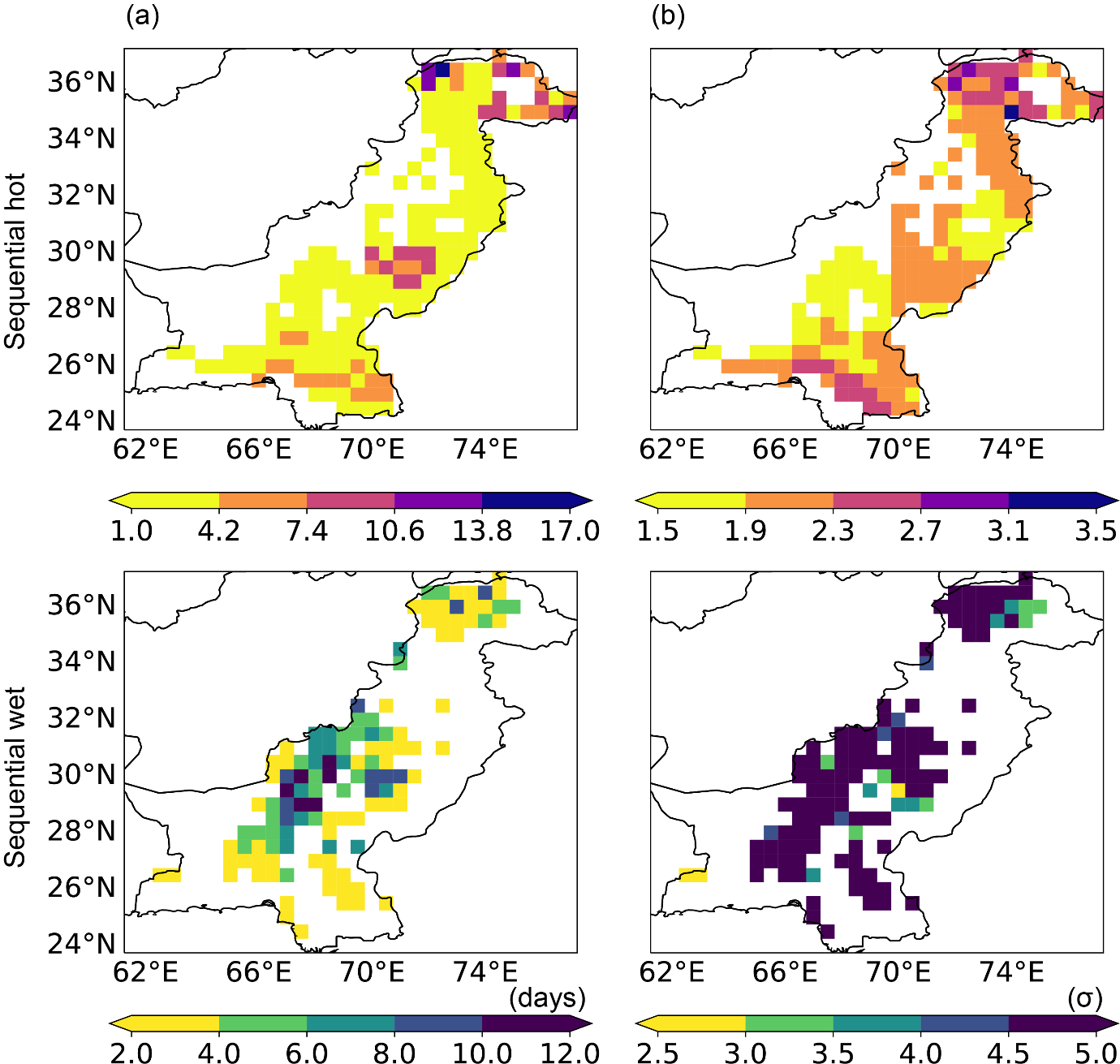


Figure S7. Number of days (a) and peak severity (b) of spring sequential hot (first row) and summer sequential wet (second row) events in Pakistan in 2022. These results are calculated based on the 98th/2nd percentile threshold.

### References

1 Ridder, N. N. *et al.* Global hotspots for the occurrence of compound events. *Nature Communications* **11**, 5956 (2020). <https://doi.org/10.1038/s41467-020-19639-3>
